# Prediction of disease genes using tissue-specified gene-gene network

**DOI:** 10.1186/1752-0509-8-S3-S3

**Published:** 2014-10-22

**Authors:** Gamage Upeksha Ganegoda, JianXin Wang, Fang-Xiang Wu, Min Li

**Affiliations:** 1 School of Information Science and Engineering, Central South University, Changsha, China; 2 College of Engineering, University of Saskatchewan, 57 Campus Dr., Saskatoon, SK Canada

## Abstract

**Background:**

Tissue specificity is an important aspect of many genetic diseases in the context of genetic disorders as the disorder affects only few tissues. Therefore tissue specificity is important in identifying disease-gene associations. Hence this paper seeks to discuss the impact of using tissue specificity in predicting new disease-gene associations and how to use tissue specificity along with phenotype information for a particular disease.

**Methods:**

In order to find out the impact of using tissue specificity for predicting new disease-gene associations, this study proposes a novel method called tissue-specified genes to construct tissues-specific gene-gene networks for different tissue samples. Subsequently, these networks are used with phenotype details to predict disease genes by using Katz method. The proposed method was compared with three other tissue-specific network construction methods in order to check its effectiveness. Furthermore, to check the possibility of using tissue-specific gene-gene network instead of generic protein-protein network at all time, the results are compared with three other methods.

**Results:**

In terms of leave-one-out cross validation, calculation of the mean enrichment and ROC curves indicate that the proposed approach outperforms existing network construction methods. Furthermore tissues-specific gene-gene networks make a more positive impact on predicting disease-gene associations than generic protein-protein interaction networks.

**Conclusions:**

In conclusion by integrating tissue-specific data it enabled prediction of known and unknown disease-gene associations for a particular disease more effectively. Hence it is better to use tissue-specific gene-gene network whenever possible. In addition the proposed method is a better way of constructing tissue-specific gene-gene networks.

## Introduction

The emerging paradigm of "network medicine" has been proposed to utilize different network-based approaches to predict essential proteins [1-4], identify protein complexes [5-8] and detect candidate genes related to different diseases [9].As methodologies progress, network medicine has the potential to capture the molecular complexity of human disease while offering computational methods to discern how such complexity controls disease manifestations, prognosis, and therapy. Up to now, different types of biological data have been used to study disease related genes and complexes [10-12]. For example, Goh K., et al., [13] constructed a network that consisted of genes associated with the same disease, while Tian W., et al., [14] combined protein and genetic interactions with gene expression correlation. Ulitsky I and Shamir R [15] also combined interactions from published networks and yeast two-hybrid experiments to identify the associations. Analyses of recent research studies, according to CIPHER [16], GeneWalker [17], PRINCE [18] and RWRH [19] highlighted the associations that were derived directly from protein interactions to more distant connections in various ways. Even though genes causing similar diseases lay close to one another in the network, these algorithms did not take into account the fact that the majority of genetic disorders tend to manifest only in a single or a few tissues [13,20]. Tissue specificity is an important aspect of many genetic diseases, reflecting the potentially different roles of proteins and pathways in diverse cell lineages. In the context of genetic disorders, even though the underlying harmful mutation can exist in all the cells in the human body, it most often wreaks havoc only in a few tissues. This tissue selectivity will appear due to the differences in the functionality of the mutated protein within these tissues, its tissue-specific interacting proteins, its abundance and the abundance of its inter-actors. Hence, the purpose of this study is to investigate whether a tissue specific network was a better representation for the actual disease-related tissue, which yields to more accurate prioritizations of the disease-gene associations.

Some research has been carried out by constructing tissue specific networks to detect diseases through the Bayesian structure learning algorithms [21]. But Bayesian structure learning algorithms had three major shortcomings, that is, the high computational cost, inefficiency in exploring qualitative knowledge, and the inability to reconstruct phenotype specific gene network. Others [22] analyzed human PPIs in a tissue-specific context, showing that many housekeeping proteins interact with highly tissue-specific proteins, which in turn implies that housekeeping proteins may have tissue-specific roles. This analysis was taken a step further by Emig and Albrecht [23] who identified the functional differences between tissues, showing that tissue-specific protein interactions are often involved in transmembrane transport and receptor activation.

This study therefore seeks to construct tissue-specific gene-gene networks for a particular query disease and try to match these networks with the similar phenotype details to predict new disease-gene associations. The novel tissue-specific gene-gene network construction method called the tissue-specified genes (TSG) method would be used to initially identify the tissues mainly affecting the query disease and secondly the gene expression details of the tissues would be used to construct tissue-specific gene-gene networks. Created tissue-specific networks would be used with the most nearest phenotype details of the query disease to predict gene-disease associations. The original Katz method has been modified and used as the primary method of prioritizing disease genes by using tissue-specific gene-gene networks. The novel tissue-specific gene-gene network construction method is described in details in the methodology section.

## Methods

### Tissue specific gene expression

Gene expression profiles have been widely used with protein interaction networks to identify protein complexes, predict protein functions, construct dynamic protein interaction networks, and discover disease-related genes [24-26]. In this research, the human body index-transcriptional profiling of tissue-specific gene expression data set was downloaded from the gene expression omnibus (GEO) for GSE 7307 series [27] to predict disease genes. The dataset consisted of a total of 677 samples, representing over 90 distinct tissue types. Normal and diseased human tissues were profiled for gene expression using the Affymetrix U133 plus 2.0 arrays. Based on the case studies which has used in this study, detailed gene-expressions of 7 tissues were selected.

### Disease-tissue relationship

The relationships between diseases and tissues were considered from the work by Lage et al [28] who estimated the association of a tissue and a disease by measuring their co-occurrence in PubMed abstracts. It has created a disease-tissue co-variation matrix of high-confidence associations of >1,000 diseases to 73 tissues.

### Selection of tissue-specific gene interaction pairs

After identifying the tissues related to each query disease gene expression, details of these tissues were downloaded from GEO in the national center for biotechnology information (NCBI) website. Using these genes expression details of each query disease, Pearson correlation coefficient (PCC) was calculated [29-31] for each gene-gene interaction in the gene-gene network.

A separate tissue specific gene-gene networks was constructed for each tissue that was related to the query disease by considering the PCC values for each gene-gene interaction. The interactions that have PCC values more than the threshold value were considered for tissue specific gene-gene network and others were removed from the gene-gene network.

### Weighted TSG network

After the creation of the tissue-specified genes (TSG) network for each tissue, each interaction was weighed by considering the relationship between gene and different phenotypes along with gene expression details of each query disease. The weight of each interaction in the novel network was calculated from equation (1).

(1) S(i,j)=α∑k=1naik ajk/ Nk+(1-α)PCC

From the first part of the equation the co-occurrence of phenotypes with less annotated genes that gave more weight than well-studied, [23] broadly-defined phenotypes are shown. Therefore in the equation, a_ik_= 1 if gene *i *has phenotype k and a_ik _= 0 otherwise, and N_k _is the number of genes involved in the specific phenotype k; and n is the total number of phenotypes. In the second part of the equation it emphasis on the tissue-specificity of the interaction by incorporating PCC value. Hence, the weight represents how each interaction in the tissue specified gene-gene network reacts to different phenotypes while considering tissue-specificity. The phenotypes used for the calculation are similar phenotypes to the query disease. The similarities between phenotypes were obtained using the matrix introduced by van Driel et al [32], who used the anatomy (A) and the disease (C) sections of the medical subject headings vocabulary (MeSH) to extract terms from online Mendelian inheritance in man (OMIM) to identify similar diseases. Finally, α ∈ 0[1] is a parameter controlling the relative importance of the phenotype vs. the PCC value.

### Construction of gene-phenotype network

To construct the gene-phenotype bipartite network for each tissue type for the specific disease the following method was used. The gene-phenotype association matrix was constructed where, $p_{i}\in R^{\left|g|\times |p_{i}\right|}$, such that (Pi)_gp _= 1 if gene *g *is associated with phenotype *p *or 0 otherwise. For the matrix the phenotypes that were selected were similar to phenotypes for the query disease. In order to find the most similar phenotypes, the text mining method MimMiner was used [32].

### Construction of phenotype-phenotype network

Separate phenotype-phenotype matrices were constructed for each query disease. To select the most similar phenotypes for the query disease the MimMiner approach was utilized [32].

### Implementation of prioritization methods on TSG network

Random Walks with Restart on Heterogeneous network [19], PRINCE [18] and ProSim [33] methods were used as prioritization methods that accepted TSG networks. During the implementation of each method the entire gene-gene network was sub divided into several tissues-specified gene-gene networks depending on the query disease. Then the algorithm was executed with each sub network separately and the final results were merged from the result of each sub network.

### Random Walks with Restart on Heterogeneous network

Random Walks with Restart on Heterogeneous network (RWRH) is an algorithm for predicting gene-disease associations proposed by Li and Patra. RWRH performs a random walk on a heterogeneous network of gene interactions and human diseases [19]. The random walk is started from a set of seed nodes, which for a phenotype p is the set of genes known to be associated with p, and gene nodes are ranked by the probability that a random walker is at a given gene, under the steady state distribution for the random walk. RWRH considers the following heterogeneous network:

$$c=\left[\begin{array}{cc} G & \lambda P\\ {\lambda P}^{T} & Q \end{array}\right]$$

where G is the entire gene-gene interactions matrix, Q is the phenotype-phenotype similarity matrix, and  λis the probability that the random walker jumps from a gene node to a phenotype node (or vice versa).

### PRINCE and ProSim

PRINCE [18] and ProSim [33] are other graph-based methods that can be thought of as a special case of RWRH. In both methods random walk is used over the protein-protein interaction network instead of the heterogeneous network. Phenotype similarity is used as the restart vector in PRINCE [18] and the combination of phenotype similarity and protein proximity is used as the restart vector for ProSim method [33]. For the research experiment PRINCE algorithm has been changed where protein-protein network is replaced by the tissue-specific gene-gene network for a particular disease. The ProSim method is changed where gene-gene network is constructed by considering three features: Pearson correlation coefficient of tissue specific gene expression details of each query disease, gene's small world clustering coefficient and subcellular localization details of each protein-protein interaction. For both methods the final equation remains same for our experiment.

### Data sources

The data was downloaded from the following data sources.

Gene-gene network: HPRD database was downloaded from [34]. The edges in the HPRD network are un-weighted. This protein-protein network was used to create the gene-gene network.

Phenotype-phenotype network: with the use of OMIM phenotypes, the similarity matrix will be calculated using the MinMiner introduced by van Driel [32].

## Results and discussion

**Construction of tissue-specific gene-gene network**Six case studies were studied, in order to measure the effectiveness of the tissue specific details of each query disease, to predict disease genes. The selected cases included; Breast Cancer (MIM: 114480), Colorectal Cancer (MIM: 114500), Prostate Cancer (MIM: 176807), Lung Cancer (MIM: 211980), Alzheimer (MIM: 104300) and Diabetes Mellitus (MIM: 125853).

In order to identify the disease-tissue associations research work carried out by [28] was used. According to the study of Lagea, et al [28] a matrix was generated computationally which showed the relationship between different tissues and diseases. Systematic analysis was done between tissue-specific gene expression and pathological manifestations in many human diseases and cancers. The diseases were systematically mapped to tissues they affect from disease-relevant literature in PubMed and used to create a disease-tissue co-variation matrix of high-confidence associations of > 1,000 diseases to 73 tissues. From the results breast cancer (MIM: 114480), ovary, prostate and skin tissues were identified as the most prominent tissues affected by the disease. For Colorectal cancer (MIM: 114500), liver, lungs and ovary tissues were responsible whiles for Diabetes mellitus (MIM: 125853), liver and pancreatic islets tissues were much prominent for the disease. Whiles for Prostate cancer (MIM: 176807) prostate and skin tissues are more prominent and for lung cancer (MIM: 211980) lung and skin tissues as well. Finally for Alzheimer disease (MIM: 104300), brain tissues are more affected from the disease.

**Figure 1:**
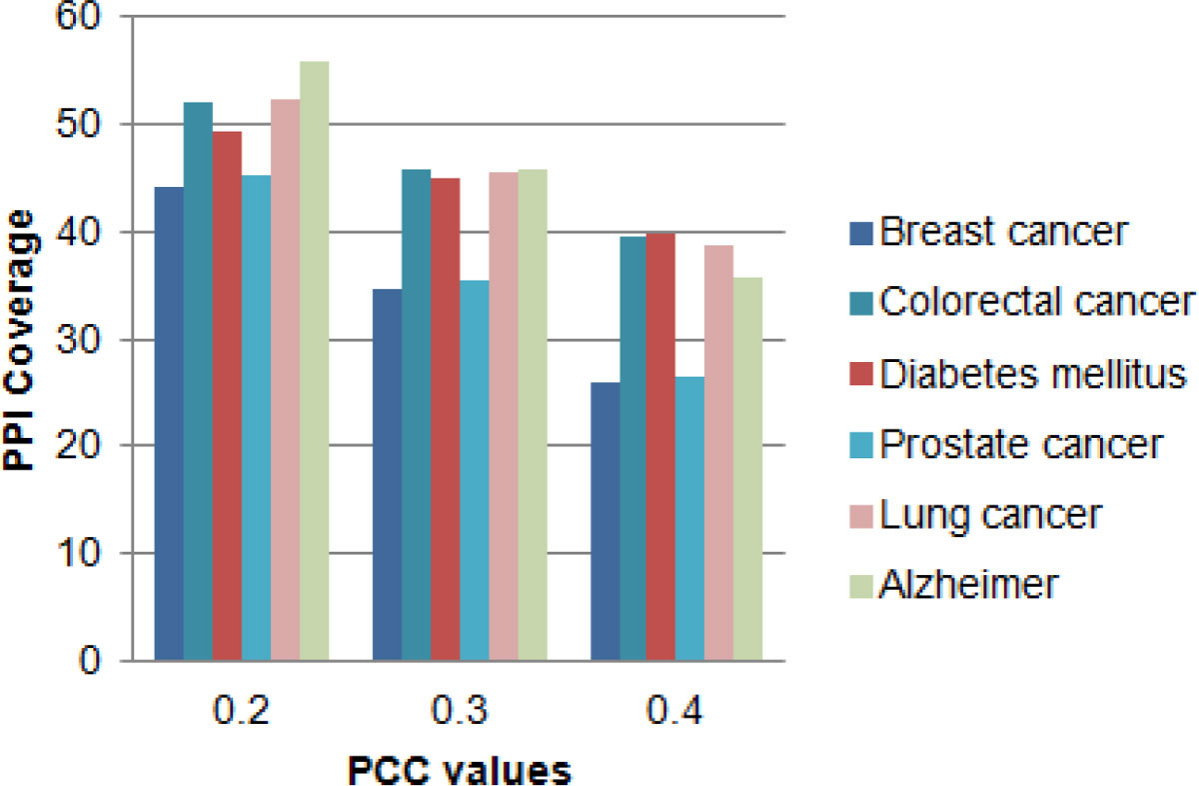
**Total coverage of the protein-protein network for different PCC values**. For breast cancer, colorectal cancer, diabetes mellitus, prostate cancer, lung cancer and Alzheimer diseases how many protein-protein interactions are covered for PCC values from 0.2 to 0.4.

After identifying the tissues for each disease, the gene expression details for each tissue sample were downloaded from the NCBI website. This consisted of human tissues measured in the Human Body Index Transcriptional. By using these genes expression details of each tissue in each query disease, PCC was calculated for the entire gene-gene network. The relationship between the PCC values and the amount of coverage within the entire gene-gene network is shown in Figure 1. From Figure 1, it was observed that more gene-gene interactions are covered if 0.2 was selected as the threshold value for PCC which unfortunately, will reduce the prediction power of the final tissue-specific gene-gene interaction network. Therefore considering the coverage and the effectiveness of predicting new disease-gene associations 0.3 was selected as the threshold value for PCC to create tissue-specific gene-gene networks.

After removing the lower PCC value gene-gene interactions from the network, all the remaining interactions were weighted using equation (1). Testing was carried out to find the best formulation between the phenotype and the tissue gene expression values. In addition, testing was repeated to check the most suitable parameter value for the equation. Testing was based on the effectiveness of predicting and detecting disease related genes from the newly created tissue-specific gene-gene network. After a series of testing the parameter α = 0.6 was finalized as the best value.

### Prediction of disease cause genes using Katz method

After constructing tissue-specific gene-gene networks, Katz method was used to check the effectiveness of the network in predicting disease genes. In order to prioritize candidate disease genes, Katz method was used because its application has been successfully tested for link prediction in social networks [35]. Furthermore, the method is based on integrating functional gene interaction networks with phenotype data and computing a measure of similarity based on walks of different lengths between gene and phenotype node pairs. Hence in this research Katz method has been used as the platform method to evaluate the performance of each method of constructing tissue-specific gene-gene networks in predicting disease genes.

By definition Katz measure is a graph-based method for finding nodes similar to a given node in a network [36]. The research done by Singh-Blom, et al [37] applied Katz method to recommending genes for a given phenotype or drug. They have introduced a Katz adjacency matrix for a heterogeneous network as:

(2) $$c=\left[\begin{array}{cc} G & P\\ P^{T} & Q \end{array}\right]$$

Let G denote the gene-gene network, let P denote the bipartite network between genes and phenotypes, and let Q denote the phenotype-phenotype network. P^T ^is the transpose matrix of P. And the final Katz score matrix S^Katz ^(C) corresponding to similarities between gene nodes and human disease nodes can be expressed as:

(3) $$S_{Hs}^{Katz}\left(c\right)=\beta P_{Hs}+\beta^{2}\left(GP_{Hs}+P_{Hs}Q_{Hs}\right)+\beta^{3}\left(PP^{T}P_{Hs}+G^{2}P_{Hs}+GP_{Hs}Q_{Hs}+P_{Hs}Q_{Hs}^{2}\right)$$

where, P_Hs _and Q_Hs _denote the gene-phenotype and phenotype-phenotype networks of humans, respectively. As well as P consist of phenotype information from multiple species. Namely: pant, worm, fly, zebrafish, E. coli, chicken, mouse and yeast phenotype information are compared with human phenotype information. β is a constant that dampens contribution from longer walks. The research study has modified the Katz method in such a way that it will accept tissue-specific gene-gene networks and it only considers gene-phenotype associations to human. Therefore the final Katz score matrix S^Katz ^(C) was calculated by considering the tissue details along with the relationship between genes and phenotypes. The equation is expressed as:

(4) $$S^{Katz}\left(c\right)=\beta P+\beta^{2}\left(GP+PQ\right)+\beta^{3}\left(PP^{T}+G^{2}P+GPQ+PQ^{2}\right)$$

where, G, P and Q denote tissue-specified gene-gene network, gene-phenotype network and phenotype-phenotype network, respectively. (Construction of gene-phenotype network and phenotype-phenotype network is explained in the method section.) The algorithm parameter β will remain same as 10^-6 ^and the number of iteration to 3 [37]. From the final matrix values we are able to predict candidate disease genes by considering the tissue-specific details.

**Figure 2:**
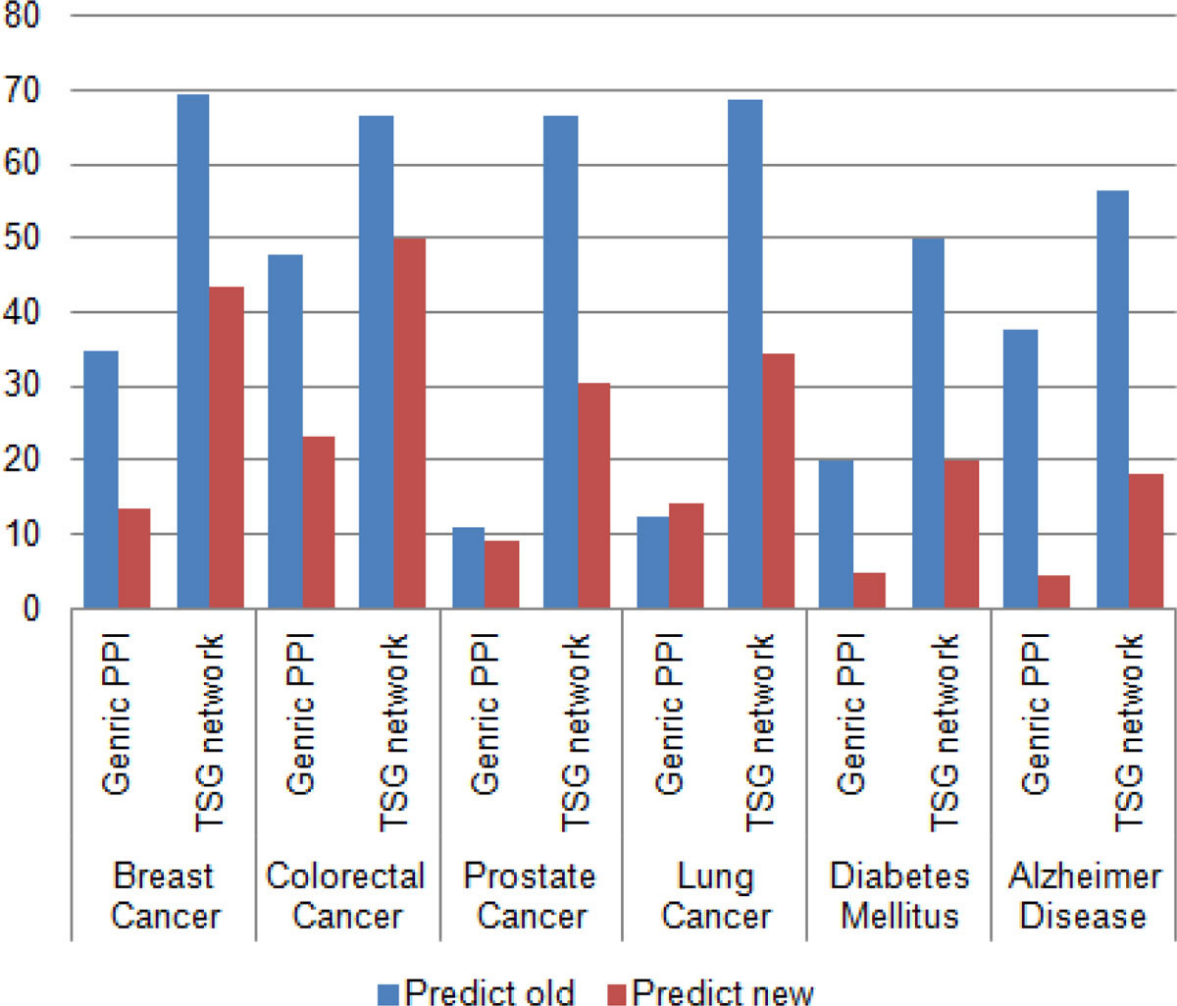
**Prediction of known and unknown disease genes between generic PPI and TSG network**. Percentage of known and unknown disease genes prediction by using generic PPI network and TSG network for breast cancer, colorectal cancer, prostate cancer, lung cancer, diabetes mellitus and Alzheimer disease.

In order to check the performance of the TSG network, it was evaluated with the generic protein-protein network by considering the effectiveness of predicting known disease genes as well as unknown disease genes. The prediction rate of known and unknown disease genes for breast cancer, colorectal cancer, diabetes mellitus, prostate cancer, lung cancer and Alzheimer is shown in Figure 2. From the result, breast cancer and colorectal cancer had a higher rate in predicting known and unknown disease genes than other diseases. Diabetes predictions showed the lowest disease genes rate as compared with others. According to the results highlighted, tissue-specific network is reacting in higher rate in predicting known and unknown disease genes for a particular disease than using generic protein-protein network.

**Figure 3:**
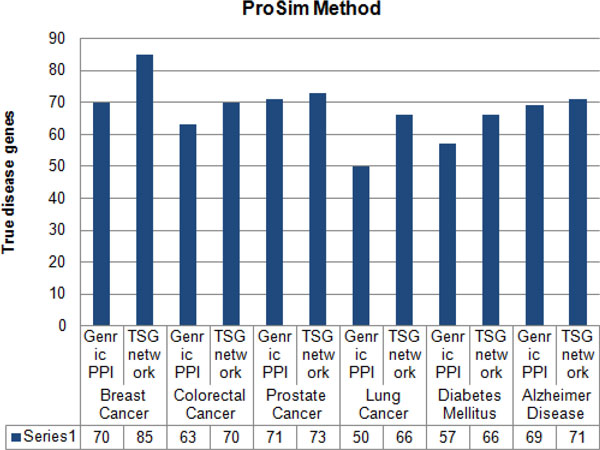
**Percentage of true disease genes detection for ProSim methods**. Percentage values of true disease genes detection by using generic PPI and TSG networks for ProSim method. Testing is carried for breast cancer, colorectal cancer, prostate cancer, lung cancer, diabetes mellitus and Alzheimer disease separately.

**Figure 4:**
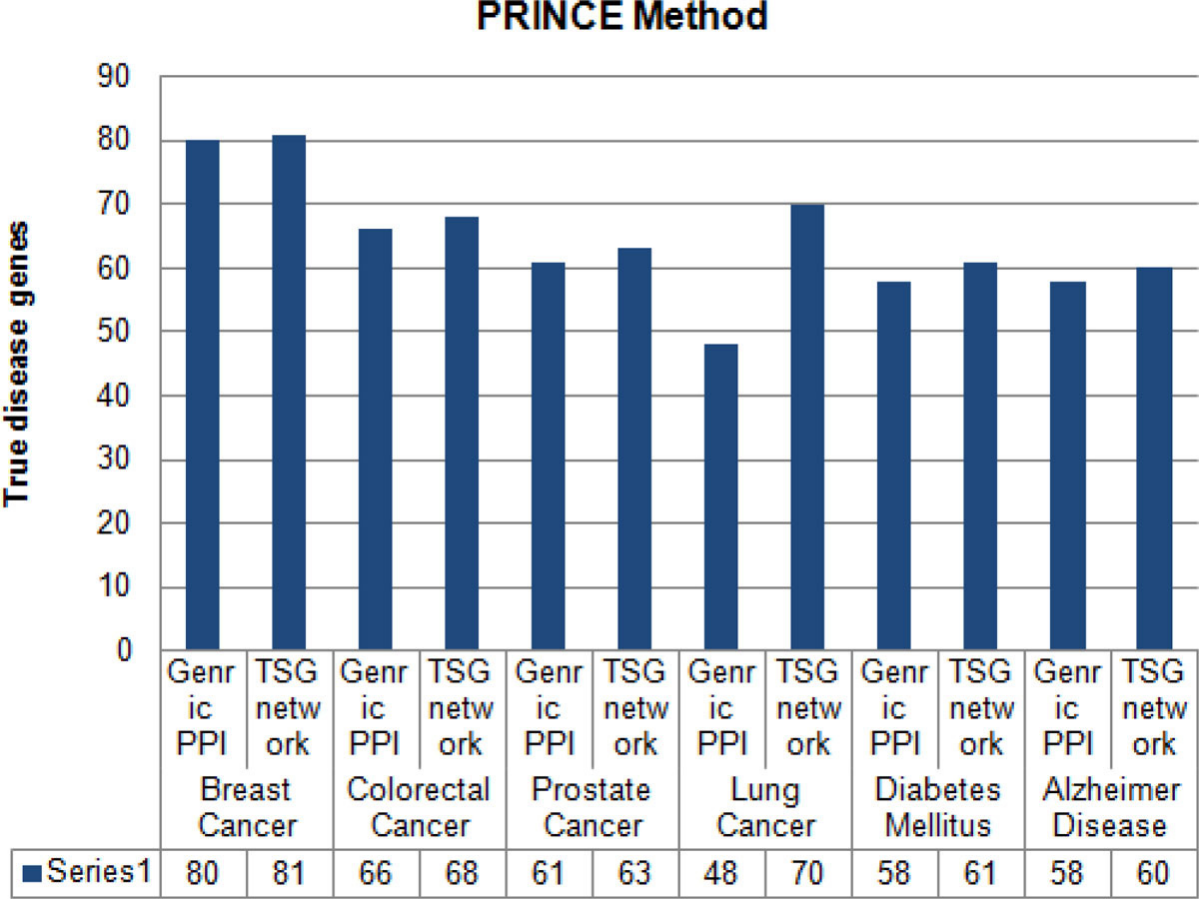
**Percentage of true disease genes detection for PRINCE methods**. Percentage values of true disease genes detection by using generic PPI and TSG networks for PRINCE method. Testing is carried for breast cancer, colorectal cancer, prostate cancer, lung cancer, diabetes mellitus and Alzheimer disease separately.

**Figure 5:**
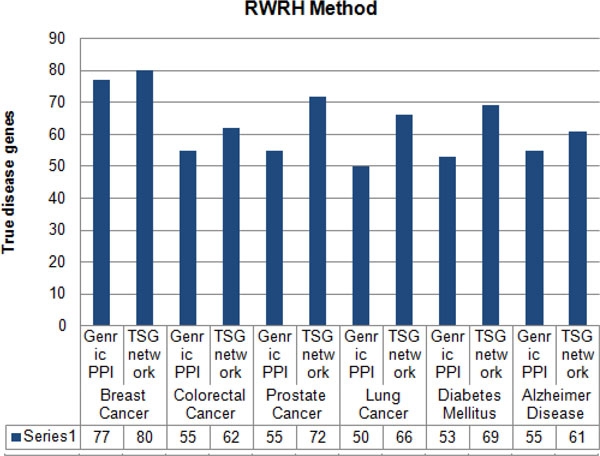
**Percentage of true disease genes detection for RWRH methods**. Percentage values of true disease genes detection by using generic PPI and TSG networks for RWRH method. Testing is carried for breast cancer, colorectal cancer, prostate cancer, lung cancer, diabetes mellitus and Alzheimer disease separately.

Furthermore, to justify the importance of using tissue-specific gene-gene network instead of generic protein-protein network for predicting and prioritizing disease genes the generated TSG network was tested with three other methods namely; ProSim [33], PRINCE [18] and RWRH [19]. Leave-one-out cross validation was carried out for each method to detect the capability of each method in predicting known disease genes at the point where generic PPI and TSG networks were used. With each cross validation trial, a single seed gene related to the query disease was removed and then each method evaluated on its success of identifying and ranking the removed seed gene. Figure 3, 4, 5 shows results of leave-one-out cross validation as in columns. According to Figure 3, 4, 5, for breast cancer by using tissue-specific gene-gene network it enables to predict true disease genes the rate of 85%, 81% and 80% for ProSim, PRINCE and RWRH methods, respectively. As well as for Alzheimer disease the values change as 71%, 60% and 61%, respectively. According to the results, we are able to conclude that by using tissue-specific gene-gene network it enables to predict more known disease genes than using a generic PPI network.

### Comparison with other network construction methods

In order to check the effectiveness of the novel method of constructing tissue-specific gene-gene network it was compared with three other methods. The methods included; tissue-specific node-removal (TS-NR) and tissue-specific edge-reweight (TS-ERW) methods designed by Magger et al [38], and BlockRank method by Jiang et al [39]. Basically node-removal tissue-specific PPI network was derived by removing from the original PPI network proteins that are not expressed in the relevant tissue and all of the edges adjacent to them [38]. The remaining edges were retained, along with their weights. In an edge-reweight tissue-specific PPI network, the confidence of each interaction represents the probability that the interaction takes place within a given tissue. This probability *rw *is calculated from the formula (5):

(5) $$\begin{array}{c} w_{ij}^{\prime }=P\left(P_{i},P_{j}\text{int}\;eract|Tissue=t\right)=\\ P\left(I_{ij}|t\right)*P\left(X\left(i,t\right)|t\right)*P\left(X\left(j,t\right)|t\right)=w_{ij}*rw^{n} \end{array}$$

where w_ij _is the original weight of the interaction and n is the number (0-2) of lowly-expressed genes in tissue t out of {Pi,Pj}. Thus, conversion of the generic PPI weight to a tissue specific PPI weight using the edge reweight method involves multiplying an edge's weighted by rw if one of its adjacent genes is not expressed in the tissue, and by rw^2 ^if neither of the edge's adjacent genes are expressed in the tissue [38]. Finally, BlockRank method [39] constructs the tissue-specific PPI network by considering only the known disease genes and the 1-order neighbors of these disease genes for a particular tissue related to each disease. Thereafter, the topology of this PPI network can be formulated as a square symmetric matrix L = (L*_ij_*) (adjacent matrix of graph G), where L*_ij _*= 1 if protein p*_i _*can interact with protein p*_j_*, and L*_ij _*= 0 otherwise. From Markov chain perspective, the PPI network can be explained by a probability transition matrix that one protein may interact with other proteins in this network with a certain degree of probability. Thus, they obtained the transition matrix of Markov model P = (P_ij_) from the adjacent matrix L as follows:

(6) $$P_{ij}=\frac{L_{ij}}{\sum_{j}L_{ij}}$$

According to the research of Jiang, et al [39] this transition matrix has been used to predict candidate disease genes. In order to check the effectiveness of each method created tissue-specific protein-protein network is forward to the tissue-specific Katz method to predict disease-gene associations for each query disease. Additional file 1 illustrates the top ten genes predicted by each method for each query disease. From the results it concludes that TSG network enables to predict more disease-gene associations than other three methods.

Evaluation process was carried out by conducting leave-one-out cross validation technique for each method. With each cross validation trial, it will hide all associations between a given gene and diseases. Therefore, validation will be done for all the known disease-gene association as well as enabling the calculation of the percentages of the true disease genes for each method. By using this evaluation method it will find out the best tissue-specific network to be used to predict and detect known disease genes for a particular disease. The percentage of true disease gene detection for each method is shown in Table 1. TSG method was able to predict disease genes; 76%, 73%, 66%, 78%, 75% and 80% for breast cancer, colorectal cancer, prostate cancer, lung cancer, diabetes mellitus and Alzheimer disease, respectively.

**Table 1:** Percentage of true disease genes for various methods.

Disease Name	TSG	NR	ERW	BlockRank
Breast Cancer	76%	42%	46%	55%
Colorectal Cancer	73%	52%	53%	61%
Prostate Cancer	66%	57%	55%	63%
Lung Cancer	78%	55%	57%	68%
Diabetes Mellitus	75%	58%	52%	66%
Alzheimer Disease	80%	68%	70%	76%

Percentage values of true disease genes detection for TSG, NR, ERW and BlockRank methods. Testing is carried for breast cancer, colorectal cancer, prostate cancer, lung cancer, diabetes mellitus and Alzheimer disease separately.

We further inspect the mean enrichment value for each method. In general, the mean enrichment formula is: enrichment = 50 / (rank), for an interval of 100 genes [40]. Based on ranking values, by using the leave-one-out cross validation process, it was possible to identify the rank of true disease genes for each method. The final results are shown in Table 2. By analyzing the results it is clear that our novel method comes first in all case studies. BlockRank method comes in the second place. For prostate cancer and diabetes mellitus NR method is in third place and for other disease ERW method comes on third.

**Table 2:** Calculation of mean enrichment for various methods.

Disease Name	TSG	NR	ERW	BlockRank
Breast Cancer	5.366	0.217	0.236	1.778
Colorectal Cancer	4.365	0.210	0.238	0.613
Prostate Cancer	1.590	0.417	0.389	1.089
Lung Cancer	10.694	1.082	1.096	3.596
Diabetes Mellitus	5.716	0.571	0.513	2.532
Alzheimer Disease	12.759	2.655	4.186	11.997

Mean enrichment values for TSG, NR, ERW and BlockRank method in the case of breast cancer, colorectal cancer, prostate cancer, lung cancer, diabetes mellitus and Alzheimer disease.

**Figure 6:**
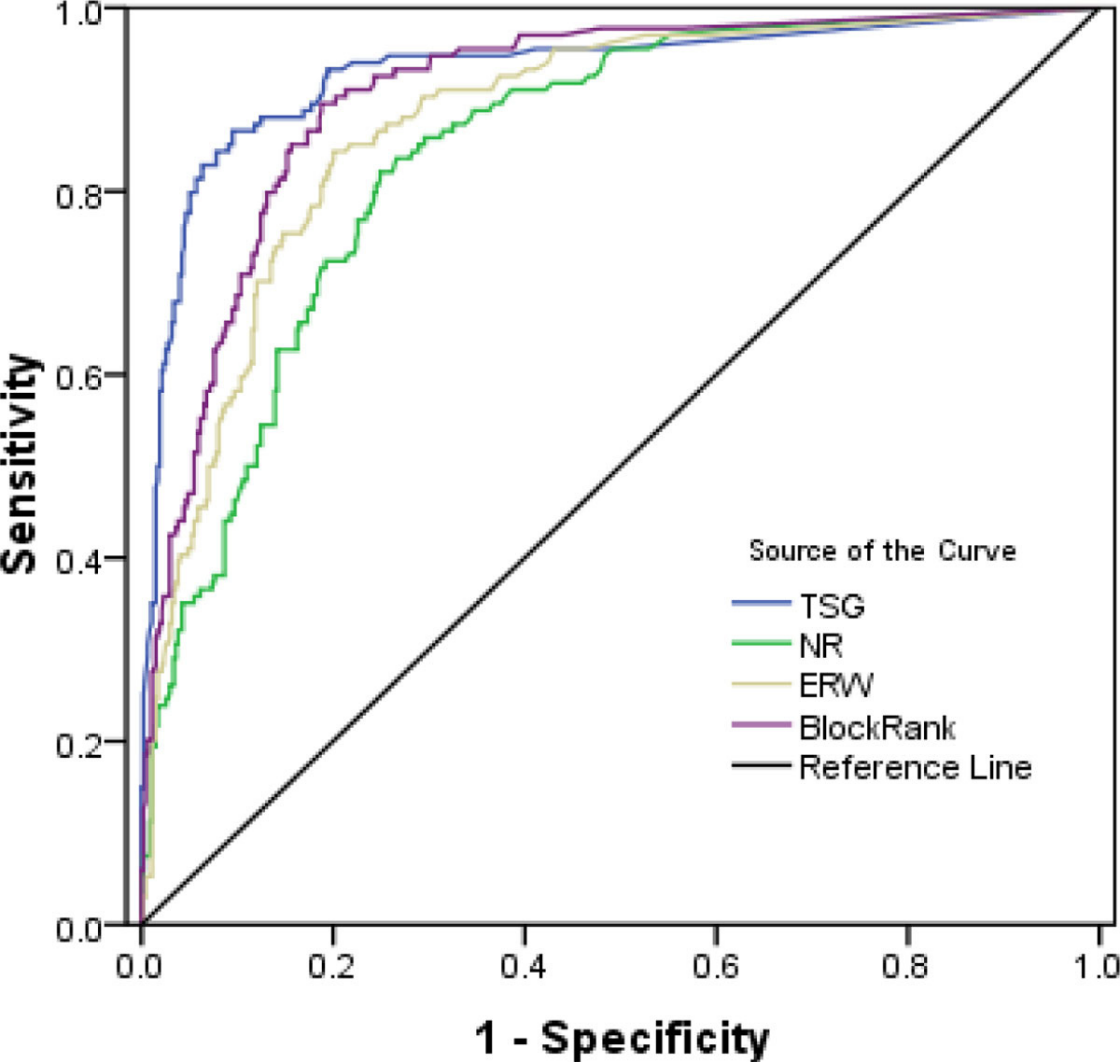
**ROC curve for Breast cancer**. Sensitivity and specificity values for TSG, NR, ERW and BlockRank network in the case of breast cancer.

Furthermore, ROC curves are drawn by considering the sensitivity and specificity, measures for each method. Sensitivity is defined as the percentage of true disease genes that are ranked above a specified threshold while specificity is defined as percentage of all non related disease genes that are ranked below a specified threshold. In other words, ROC values can be interpreted as a plot of the frequency of the disease genes above the threshold versus the frequency of disease genes below the threshold, where the threshold is a specific position in the ranking. Thus it enables to calculate the sensitivity and specificity for each case. In this scenario top 200 genes were taken into consideration. Hence the threshold value is set as 200 for the study. For breast cancer TSG method had the highest area coverage in ROC curve as illustrate in Figure 6. ROC curves for other case studies are given in additional file 2.

By considering the results, tissue-specific gene-gene network predicts more new disease genes than the generic protein-protein interaction network. By using the TSG method it is predicting that *NME1, MSH2, RAF1, HDAC1 *genes in ovary, prostate and skin tissues cause breast cancer disease [41-43]. As well as *STK11, HNF1A, TSG101, KPNA2, MDM2, APEX1 *genes in lungs, liver and ovary tissues are also tumor progression genes for colorectal cancer [44-46]. Furthermore *INS, INSR, RXRA, MAPK8 *genes in liver and pancreatic islets tissues is effective for diabetes mellitus disease. For Alzheimer disease *HTT, PRNP, KAT5 *genes in brain tissues are stimulating the disease [47-51]. *TP53, AKT1, BARD, MUC4 *genes [52-54] in lung and skin tissues are effective for lung cancer and *TP53, NTRK1, BARD1, MDM4, E2F1 and CASP8 *genes [55-57] in prostate and skin tissues are tumor progression genes for prostate cancer. As well as for breast cancer by using the TSG method it enables to detect some genes that help for breast cancer recovery. Namely: *MDM4, SMARCA4, E2F1 *and *SMAD3 *[58,59] are some of the tumor suppression genes that help for drug discovery and therapy. *BID *and *PEA15 *are two genes [60,61] that detect in lung cancer that help for drug discovery and therapy.

## Conclusions

The purpose of the research was to find out the importance of using tissue-specific details in predicting disease-gene associations and to check whether it is appropriate to use tissue-specific gene-gene network instead of generic protein-protein network at all time in predicting disease-gene associations.

A novel method was therefore proposed to construct tissue-specific gene-gene networks. The performance of the proposed method was evaluated and compared with three other methods, NR, ERW and BlockRank. The proposed method outperforms above mentioned methods. At the same time experiments were carried out to check the effectiveness of using tissue-specific gene-gene networks instead of generic protein-protein networks to predict disease-gene associations. With the results it was clear that tissue-specific gene-gene networks performed better than any other methods. It was also able to predict more known and new disease-gene associations for a particular disease. Hence the study was able to omit the use of generic protein-protein networks in predicting disease-gene associations. Even though it outperforms existing methods considered, further experiments need to be carried out to tune its performance in prioritizing candidate genes.

## Competing interests

The authors declare that they have no competing interests.

## Authors' contributions

GUG obtained the protein-protein interaction data and tissue-specific details, developed the method and analysed the results. GUG and JXW designed the method. GUG, JXW and ML discussed extensively about the study and drafted the manuscript together. JXW, FXW and ML participated in revising the draft. All authors have read and approved the manuscript.

## Supplementary Material

Additional File 1 Click here for file

Additional File 2 Click here for file

## Declarations

The publication costs for this article were funded by the National Natural Science Foundation of China under Grant No.61232001.

This article has been published as part of *BMC Systems Biology *Volume 8 Supplement 3, 2014: IEEE International Conference on Bioinformatics and Biomedicine (BIBM 2013): Systems Biology Approaches to Biomedicine. The full contents of the supplement are available online at http://www.biomedcentral.com/bmcsystbiol/supplements/8/S3.
