# Supplementary material for: Prediction of disease genes using tissue-specified gene-gene network
Source: BMC Syst Biol. 2014 Oct 22;8(Suppl 3):S3. doi: 10.1186/1752-0509-8-S3-S3 (PMC4243117; doi:10.1186/1752-0509-8-S3-S3)
Supplement: Additional File 2 [file 1752-0509-8-S3-S3-S2.pdf]

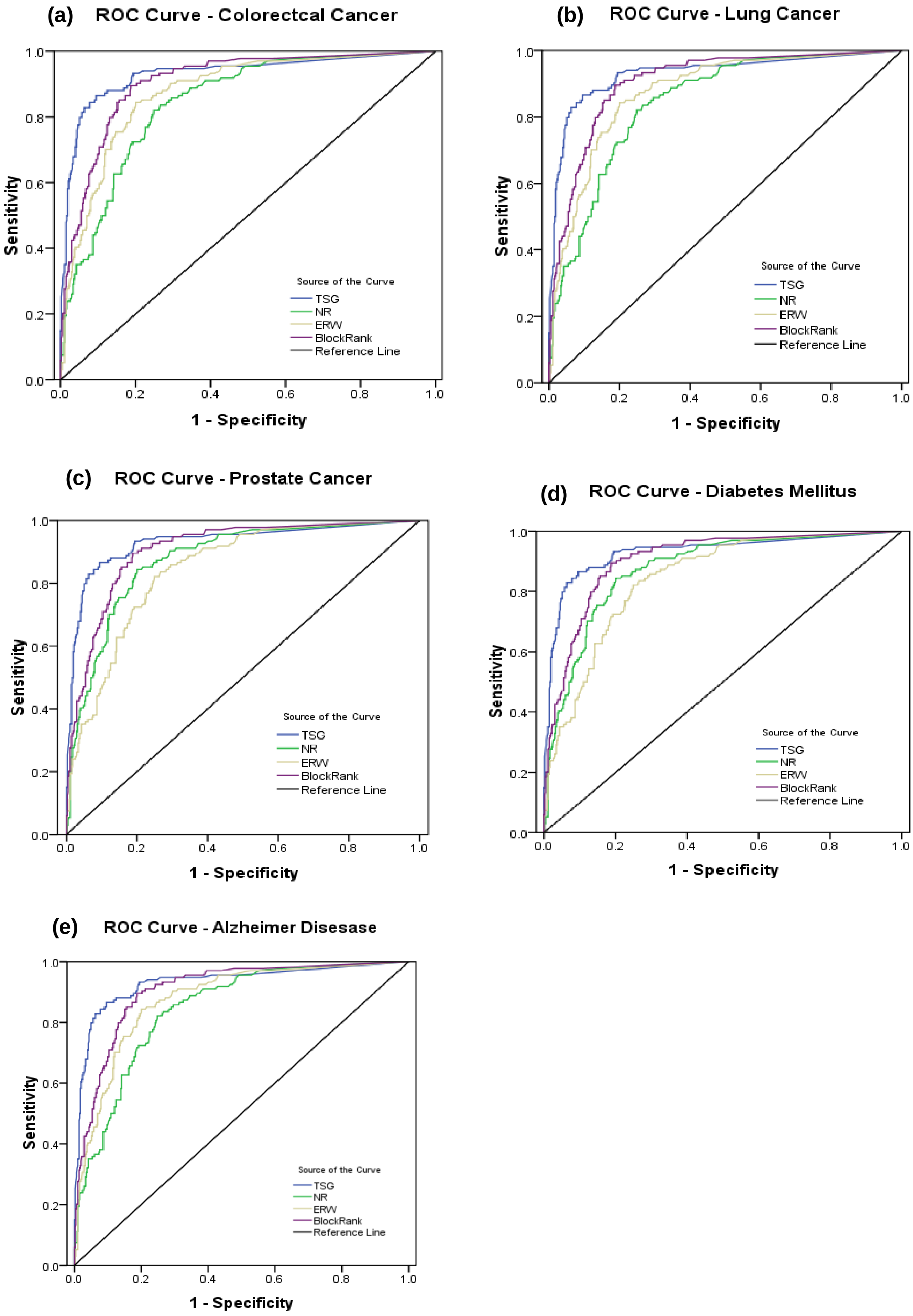
(a) ROC Curve - Colorectcal Cancer
Sensitivity
1 - Specificity
Source of the Curve
TSG
NR
ERW
BlockRank
Reference Line
(b) ROC Curve - Lung Cancer
Sensitivity
1 - Specificity
Source of the Curve
TSG
NR
ERW
BlockRank
Reference Line
(c) ROC Curve - Prostate Cancer
Sensitivity
1 - Specificity
Source of the Curve
TSG
NR
ERW
BlockRank
Reference Line
(d) ROC Curve - Diabetes Mellitus
Sensitivity
1 - Specificity
Source of the Curve
TSG
NR
ERW
BlockRank
Reference Line
(e) ROC Curve - Alzheimer Disesase
Sensitivity
1 - Specificity
Source of the Curve
TSG
NR
ERW
BlockRank
Reference Line
